# Triboelectric Charging of Particles, an Ongoing Matter: From the Early Onset of Planet Formation to Assembling Crystals

**DOI:** 10.1021/acsomega.2c05629

**Published:** 2022-10-17

**Authors:** Kai Sotthewes, Han J. G. E. Gardeniers, Gert Desmet, Ignaas S. M. Jimidar

**Affiliations:** † Physics of Interfaces and Nanomaterials, MESA+ Institute for Nanotechnology, University of Twente, P.O. Box 217, 7500AEEnschede, The Netherlands; ‡ Mesoscale Chemical Systems, MESA+ Institute for Nanotechnology, University of Twente, P.O. Box 217, 7500AEEnschede, The Netherlands; ¶ Department of Chemical Engineering CHIS, Vrije Universiteit Brussel, Brussels1050, Belgium

## Abstract

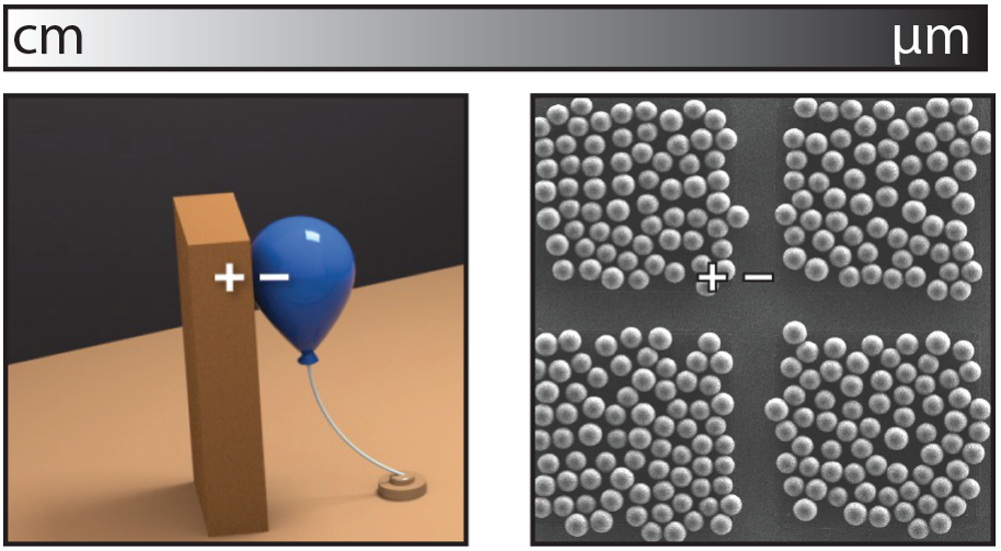

Triboelectrification is the spontaneous charging of two bodies when released from contact. Even though its manifestation is commonplace, in for instance triboelectric nanogenerators, scientists find the tribocharging mechanism a mystery. The primary aim of this mini-review is to provide an overview of different tribocharging concepts that have been applied to study and realize the formation of ordered stable structures using different objects on various length scales. Relevance spans from materials to planet formations. Especially, dry assembly methods of particles of different shapes based on tribocharging to obtain crystal structures or monolayers are considered. In addition, the current technology employed to examine tribocharging in (semi)dry environments is discussed as well as the relevant forces playing a role in the assembly process. In brief, this mini-review is expected to provide a better understanding of tribocharging in assembling objects on the nano- and micrometer scales.

## 1 Introduction

Particle assembly, either spontaneous or by human intervention, into patterns or structures offers a rich complexity to feed the perpetual curiosity of scientists to study this multiscale problem relevant to the formation of colloidal crystals or planets. Assembling building blocks into ordered structures hinges on balancing distinct interactions, including gravity, capillary, van der Waals, hydrophobic, and electrostatic interactions.^1^ The latter may result from the triboelectric charging phenomenon, which has been ubiquitous since antiquity but is often wrongly credited to the Greek philosopher Thales. There is in fact no evidence that Thales even performed the experiments typically described in many reports, but Plato wrote about electrostatic charging in his Timaeus.^2^

**Figure 1:**
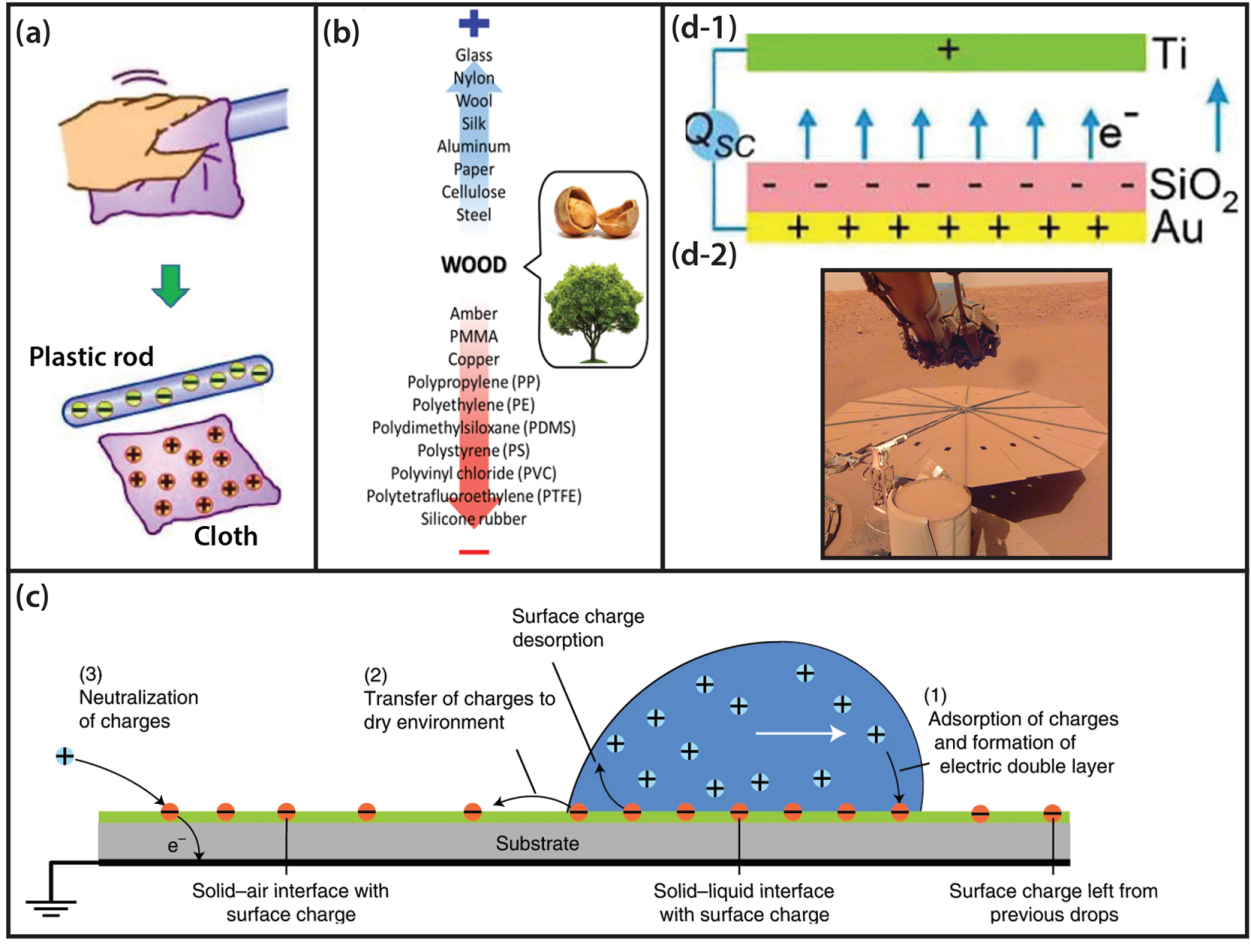
(a) Illustration of the tribocharging phenomenon between a piece of cloth and a plastic rod. (b) An exemplary triboelectric series ranking the material’s tendency to gain a certain polarity. Wood does not tend to tribocharge due to the presence of lignin. (c) A sliding drop exchange charge with a polymer film. (d-1) The charge transfer *Q*_SC_ in a TENG. (d-2) A dusty solar panel of NASA’s InSight Mars lander. (a, d-1) Reproduced with permission from ref (5). Copyright 2019 Elsevier. (b) Reproduced with permission from ref (8). Copyright 2020 American Chemical Society. (c) Reproduced with permission from ref (9). Copyright 2022 Springer Nature. (d-2) Reproduced with permission from ref (10). Copyright 2022 Courtesy NASA/JPL-Caltech.

Tribocharging is an interfacial process in which two surfaces exchange electrical charges when rubbed past each other (Figure 1a). Consequently, the bodies may gain opposite polarity, leading to the onset of an electrostatic attraction. Another term frequently introduced in the literature for the charge exchange process between two surfaces is contact electrification, distinguishing between rubbing (triboelectric charging) and touching or collision (contact charging) types of contact. However, this distinction may be misleading, as apparent macroscopic touching can sometimes in reality be considered as localized rubbing of nano asperities on the surface.^3^ This emphasizes the conundrum one faces when dealing with this mesoscale phenomenon.

The effects of tribocharging are commonly known to bring joy when we play a simple game of rubbing a balloon across our hair or decorating a cat with Styrofoam. These elementary examples would deceivingly give a novice the illusion that it is relatively simple to comprehend this phenomenon, yet the truth is that our fundamental understanding of electrostatic charging may still be in its infancy. For centuries, the scientific community endeavored to unravel this mysterious phenomenon, but currently, there is no consensus among scientists on the exact underlying tribocharging mechanism, particularly when it concerns insulators. A few tribocharging mechanisms proposed so far include electron transfer, material transfer, ion transfer, density of states, mechanochemistry, and water layers.^4,5^ On the other hand, the empirically established triboelectric series is a guide for scientists in the modern days to determine in which direction the charge will be transferred during the rubbing process (Figure 1b). The triboelectric series rank the materials based on an asymmetry in property such that materials ranked on top of the series charge positively, while materials at the tail gain a negative charge. A critical remark here is that the triboelectric series lacks every fundamental basis as deviating results from the prediction are often obtained. The former is highlighted explicitly by studies that show that identical materials also charge after being in contact. Some scientists are even conjecturing that the triboelectric series are ranked according to the hydrophilicity of the substrate.^3,6^ They assume that hydrophilic materials charge more positively, while materials with increasing hydrophobicity are more found toward the tail of the series. The latter coincides with the notion that OH^–^ ions present in the atmosphere or a basic environment act as charge carriers that prefer adhering to hydrophobic surfaces, charging the surface more negatively,^6^ as depicted for example in Figure 1c. On the other hand, Shin et al. recently introduced a novel quantitative triboelectric series based on material properties, such as density, specific heat, thermal conductivity and the Seebeck coefficient.^7^ However, this model also fails to predict the direction of charge transfer between surfaces of identical materials.

**Figure 2:**
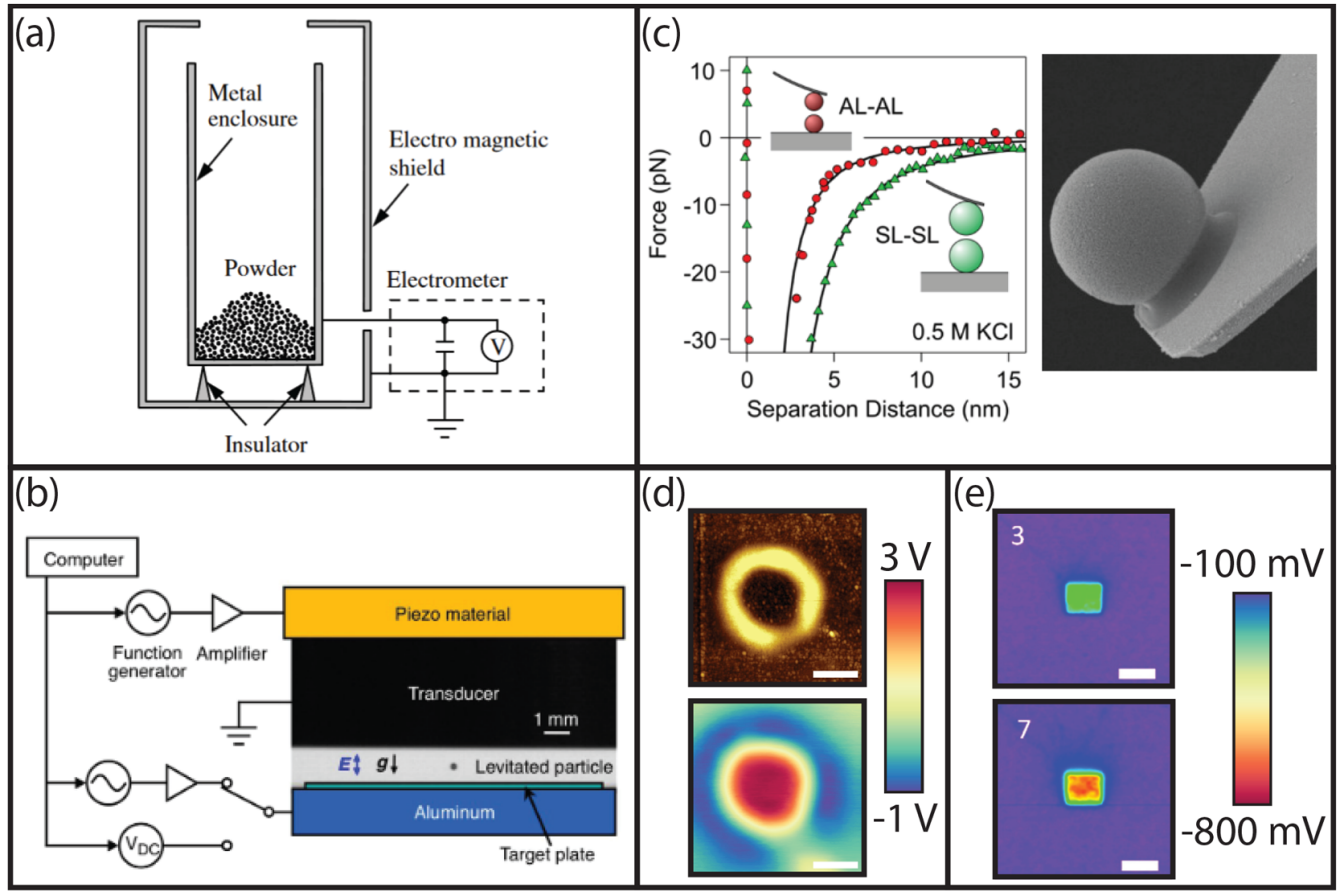
Several experimental studies to characterize triboelectric charge. (a) Faraday-cup setup. (b) A submillimeter particle is acoustically levitated between a grounded ultrasonic transducer and a sound-reflecting target plate. A piece of aluminum underneath the target plate is connected to an ac or dc voltage source. The particle was back lit and filmed from the side with a high-speed camera. The whole setup is enclosed in a chamber to control the ambient gas. (c, left) An AFM force–distance curve obtained for a symmetric system; AL = amidine latex, and SL = sulfate latex. (c, right) SEM image of a colloidal AFM probe (colloid diameter 10 μm). (d) Topographic image (1 × 1 μm; scale bar = 250 nm) and the simultaneously obtained potential map of an impact crater created by an impacting silica particle on the CF_*x*_ coated surface. (e) Triboelectric charge accumulation measured with the KPFM on the SiO_2_ surface with the increase of the number of repeated rubbing at the same area (20 × 20 μm; scale bar = 5 μm). (a) Reproduced with permission from ref (4). Copyright 2010 Elsevier. (b) Reproduced with permission from ref (6). Copyright 2018 American Physical Society. (c) Reproduced with permission from ref (14). Copyright 2015 American Chemical Society. (d) Reproduced with permission from ref (12). Copyright 2022 Elsevier. (e) Reproduced with permission from ref (15). Copyright 2013 American Chemical Society.

Despite that the scientific community is still puzzled by its exact mechanism, the concept of tribocharging has already been leveraged for decades in applications, such as photocopying, electrospraying, electrochemistry, separation of particles, lignin as antistatic agents in polymers (CF. Figure 1b), and, more recently, as triboelectric nanogenerators (TENGs) employed as sensors, or energy harvesters. For example, these energy harvesters may benefit from recent findings that sliding droplets generate electrical charges spontaneously on hydrophobic surfaces as shown in Figure 1c.^9^ However, tribocharging can also inflict damage or reduce application efficiency when particles stick on fluidized beds’ walls and dust particles cover solar panels on Earth or Mars (Figure 1d-2). Tribocharging is also relevant to processes such as agglomeration in granular materials, powder blending in the pharmaceutical industry, conveyor belts, and crystal formation.

Therefore, the present contribution focuses explicitly on the tribocharging of beads ranging from the millimeter size down to the size of colloidal particles. To this end, the different interaction forces relevant to each scale are first qualitatively discussed. A key part of this mini-review sheds light on state-of-the-art technology employed to examine tribocharging. In addition, we cover previous reports that used dry assembly methods based on tribocharging to obtain crystal structures or monolayers. Next, we touch upon tribocharging of identical materials, including induced polarization, applicable to agglomeration or cluster formation in granular systems. Finally, we provide a summary and address various points for future exploration to solve the open questions in particle tribocharging.

## 2 Surface Interaction Forces

Classical mechanics dictates that the dynamics of all bodies depend on the forces acting on them in conjunction with their mass *m*. As far as particulate matter is concerned, the forces acting on the particles can be characterized as long-range and short-range forces.

Gravity is commonly known to pull on all bodies, and for a spherical body with radius *R*, its magnitude scales as ∝ *gR*^3^, where *g* denotes the acceleration of gravity. Another force that acts on particles carrying charge *q* is the Coulomb force *F*_C_. When charged particles are subjected to an electric field E⃗, the Coulomb force equals *F*_C_ = *qE*. Charged particles can also induce Coulomb interactions with each other. In this case . Consequently, it is inferred from elementary physics that like-charged particles repel each other, whereas oppositely charged bodies attract. However, as we will address in section 5, it is plausible that under certain conditions, an electrostatic attraction exists between beads that carries the same polarity on their surface. Gravity and Coulomb forces dominate the long-range interactions among relatively large spherical particles.

On the other hand, as the size of the beads decreases, short-range cohesion forces could become more prominent, causing particles to stick to each other and form aggregates. Potential sources contributing to cohesive interactions include van der Waals, contact mechanics, and capillary forces. The latter is mainly prevalent when it involves hydrophilic particles, as a liquid meniscus is formed between touching particles covered with an adsorbed water layer. The van der Waals force originates from electromagnetic interactions between neutral molecular dipoles, while the contact mechanics force stemming from the van der Waals interactions accounts for the elastic deformation present at the interface of two contacting particles, e.g., JKR Theory.^11^ All these cohesive interaction forces *F*_coh_ scale as γ*R*, in which γ denotes the interfacial energy that depends on the material and temperature. For example in the case of silicates, γ is typical of the order of tens of milli-Newtons m^–1^, implying that for a silica particle with a radius of 5 μm, the cohesive interactions are of the order of μN, whereas the gravity is only on the order of tens of pN. Note that in practice, the cohesive forces between bodies are significantly reduced, due to the presence of micro or nano asperities on their surface.^11,12^

From the preceding discussion, it can be inferred that only the Coulomb interactions may be repulsive in nature and that in comparison to the cohesive forces *F*_coh_, gravity can be neglected as the size of the beads is reduced. A caveat is that the particles exhibit surface roughness, reducing the cohesive interactions by at least 2 orders of magnitude. The granular Bond number *Bo*_g_ is a measure of the strength of the cohesive interactions with respect to gravity, i.e., *Bo*_g_ = *F*_coh_/Gravity, implying that for fine or ultrafine cohesive powder particles *Bo*_g_ > 1, whereas for large beads *Bo*_g_ < 1. It is noteworthy that when the interaction forces cause particles to stick to other surfaces, e.g., flat substrates, they are referred to as adhesion forces. The interested reader is kindly referred to respective studies for a detailed discussion of interaction forces.^11,12^

## 3 Characterizing Triboelectric Charging

One of the techniques predominantly employed to measure the electrical charge on millimeter- or sub-millimeter-sized particles or any other type of surface^13^ are the Faraday cup and Faraday cage or adaptions thereof. In the Faraday cage setup depicted in Figure 2a, the particles are placed in a metal enclosure that is isolated, while charge is induced in the inner metal wall of the enclosed system. This is induced charge is measured trough the electrical connection with an electrometer, i.e., the current passing through a connected electrometer is recorded. A deficit of the Faraday cup is that when an ensemble of submillimeter particles is flown through the cup, particle–particle or particle–wall interactions before entering the Faraday system can not be prevented from happening, hindering the interpretation and lowering the accuracy of the measured charge on the particles significantly.

Another route one may take to study tribocharging effects under controlled conditions, e.g., constant humidity, is forcing a single particle to impact on a target plate. Recently, Lee et al.^6^ designed a sophisticated setup shown in Figure 2b where the material-independent acoustic levitation and high-speed imaging are combined to study the dynamics and charge transfer of multiple collisions of single submillimeter microspheres with a diameter of ≈200 μm against a target in a controlled manner. The rationale of this method is that by sequentially applying acoustic levitation wherein a particle is first trapped and subsequently released, a collision is initiated after which the bouncing particle is immediately captured by the acoustic trap. This strategy ensures that exactly one collision occurs between the particle and target. The net charge *q* on the particle is extracted by tracking the response of the acoustically trapped particle on the application of an oscillating electric field, allowing for measuring the evolution of the net charge *q* induced on the particles after multiple collisions. However, if one would be interested in measuring the maximum amount of charge that can be exchanged between the particle and target, this method is flawed, as the particle inevitably rotates so that a new spot on the particle surface collides with the target each time. Therefore, measuring the maximum amount of charge can become a daunting task requiring running the experiment for days. Another significant drawback of these methods is that they cannot be employed to study the tribocharging dynamics at high relative humidity conditions or of relatively small microparticles impacting against a target plate. Owing to the interaction forces elaborated on in section 2, impacting particles will not bounce back from but rather stick to the targets, as outlined in a detailed study on particle impacts against target electrodes.^16^

With the introduction of the colloidal probe technique, scientists gained access to an excellent instrument to characterize various interactions of microspheres or colloids with other bodies using atomic force microscopy (AFM) (cf. Figure 2c). The method has become state-of-the-art when measuring friction and surface interaction forces, including long-range electrostatic interactions,^14,17^ on the micro- and nanoscales. Different contact geometries can be studied, such as colloids on flat-surfaces and colloid–colloid interactions. In Figure 2c, the difference in electrostatic interaction is observed between two distinct types of latex particles. However, only a few studies have applied this technique to study tribocharging by pressing a probe against a substrate and quantifying the electrostatic force acting on the probe. Tapping the colloidal probe on the same spot addresses the issue of measuring the saturation charge that can be exchanged between a probe and a target under controlled conditions, but the downside is that one is left in the dark in which direction charge transfer occurred, i.e., the polarity of the involved surfaces is unknown.

To this end, Kelvin probe force microscopy (KPFM) measurements, also executed with AFM, are currently scientists’ best bet to characterize the surface potential and polarity of micro- and nanoparticles with high spatial resolution. This technique was initially developed to characterize the local contact potential difference (CPD) between a conductive tip and a metal surface in a closed electrical circuit, thus mapping the work function. On the other hand, the KPFM is also becoming more and more common to identify the surface potential of dielectric particles^12,18^ and insulating substrates using a conductive tip (cf. Figure 2d).^16,19^ Furthermore, experiments are being reported in which the conductive tip is first rubbed on an area of the insulator substrates, e.g., SiO_2_, in contact mode. Subsequently, a larger area is scanned to examine the evolution of charge diffusion across the surface on the nanoscale (cf. Figure 2e).^15,20^ However, it remains highly complex to retrieve quantitative data on the exact amount of charge present on nonconducting surfaces. As a consequence, only qualitative data, such as the surface’s polarity, after the tribocharging process can be acquired. It has been attempted by Xu et al. to resolve the qualitative KPFM data to calculate the number of charges present on semiconductor surfaces,^21^ but for insulators, such methods are unprecedented.

## 4 Self- and Directed Assembly of Beads on Multiscale: Particles and Substrate Interactions

As the miniaturized device era emerged, an overwhelming diversity of (self-)assembly techniques have been proposed in the literature to manufacture ordered micro- and nanoparticle arrays used as, e.g., photonic films, structural colors, and colloidal lithography masks. To produce these assembled structures successfully, tuning the particle–substrate and interparticle interactions is paramount.^1^ Wet assembly techniques in which particles are dispersed in solvents are usually the preferred choice, as they give the advantage of circumventing and controlling the cohesive interactions (cf. section 2) during assembly, e.g., tuning van der Waals interactions, electrostatic repulsion, or attraction, between the particles and substrates. However, the attained structures’ quality in this case predominantly hinges on highly optimized assembly conditions, e.g., solvent evaporation rate, surface wettability, temperature, and acidity. Any slight deviations from the optimum may lead to tremendous defects, such as cracks in the assembled structures.

On the other hand, dry assembly techniques may offer some benefits as they could potentially be faster, more amenable to automation, and have a higher tolerance for small particle dispersity. Nevertheless, it is incredibly challenging to manipulate and control the interaction forces that cause particles to stick, consequently impairing the assembly of ordered structures under ambient conditions. Therefore, exploiting dry assembly techniques, particularly electrostatic-driven assembly, is a feat hardly undertaken by scientists. However, a limited number of studies applied the tribocharging mechanism with great success to study particle (self-)assembly.

For example, the Whitesides’ group used a chemically directed tribocharging mechanism entailling the transfer of mobile ions (=counterpart of covalently bound ions) from one surface to another upon contact to assemble 3D ordered microstructures under dry conditions.^26^ The microstructures are created by agitating large (200 μm) and a substantial excess (at least a factor of 10) of smaller polystyrene spheres (5 μm) in an aluminum dish, whereby the surface of the small and large microspheres had distinct functional groups. When the particles came in contact, the large and small spheres gained opposite polarity, inducing an electrostatic attraction between them. As a consequence, the surface of the large spheres was decorated with a closely packed ordering of small spheres. The latter is remarkable as it indicates that the electrostatic attraction between the large and small spheres must be more substantial than the repulsion among the closely packed small spheres carrying the same polarity. Another explanation is that the attractive induced polarization (cf. section 5)^27^ among the small spheres enhances the stability of these equilibrium structures. It is important to comment that if these experiments were performed on a substrate carrying an insulator layer instead, the particles would stick on the dish due to tribocharging, failing to assemble into the desired structure. In addition, it can be argued that, since a massive excess of smaller beads was needed, the method can be considered inefficient due to substantial material loss. The latter is generally the norm for dry assembly methods, whereas the dosage of particles can be better controlled in a wet state.

**Figure 3:**
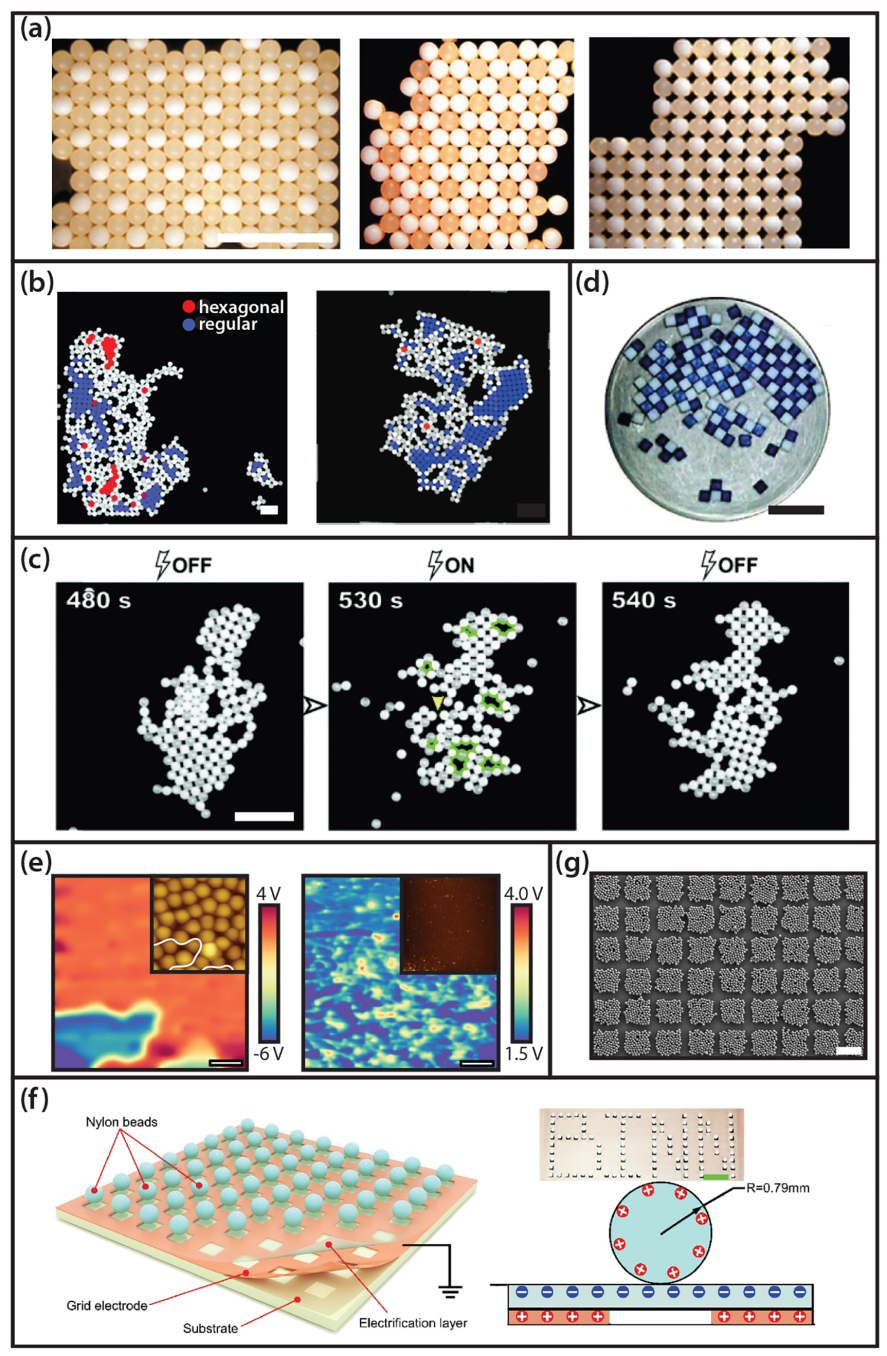
(a) Electrostatic assembly of macroscopic crystals comprising a binary mixture of polymer beads; scale bar = 20 mm. Granular lattice structures self-assembled after agitating a binary mixture of beads with (b, left) one, or (b, right) two conductive plate(s); scale bar = 10 mm. (c) A sequence of the lattice structures after turning the applied electric field on and off between the two conductive plates; scale bar = 10 mm. (d) Assembly of agitated cubic objects; scale bar = 20 mm. KPFM measurements performed after vibration experiments on (e, left) silica microspheres and (e, right) fluorocarbon-coated silicon substrate. Insets represent the corresponding topography scans; scale bar = 5 μm (f) Schematic representation of the electrostatic templated self-assembly of macro-sized spheres; scale bar = 10 mm. (g) Segregation of rubbing-induced silica micropsheres on fluorocarbon-coated glass substrate; scale bar = 50 μm. (a) Reproduced with permission from ref (22). Copyright 2003 Springer Nature. (b, c) Reproduced with permission from ref (23). Copyright 2021 Royal Society of Chemistry. (d) Reprinted with permission from ref (24). Copyright 2012 Royal Society of Chemistry. (e) Reproduced with permission from ref (12). Copyright 2022 Royal Society of Chemistry. (f) Reproduced with permission from ref (25). Copyright 2018, American Chemical Society. (g) Reproduced with permission from ref (19). Copyright 2020, American Chemical Society.

Instead of using a chemically directed assembly approach, a series of studies utilized two distinct millimeter-sized polymer spheres based on their position in the triboelectric series to attain 2D binary granular crystals by laterally agitating these spheres with a peak acceleration of ≈0.02*g* inside a container coated various types of material (Figure 3a).^22−24^ Note that these experiments represent a complex three-material tribocharged system, as charge exchange can occur between the two different types of spheres, including the bottom and walls of the container. That is, tribocharging of the agitated beads occurs as the beads roll or slide across the container, hitting the container walls and contacting other beads. Despite the inherent complexity of the problem, the results seem promising as steady-state 2D granular lattices with square, pentagonal or hexagonal symmetry are achieved. The type of symmetry depends on the ratio of the distinct particles. Of course, for the formation of crystals spanning a large area, it is essential that the beads acquire opposite charges during agitation, and the electrostatic attraction between the beads must outweigh any interaction between the beads and the container. The dynamics of the crystal formation is underpinned by the existing competition between the charging kinetics of the different materials with each other, i.e., the electrostatic attraction, as well as the particles’ kinetic energy, determine if a certain packing symmetry is attained. Cademartiri et al. measured that these ordered crystal structures contain no net charge,^24^ whereas other studies found no such evidence.^22,23^ The latter is to be expected as the beads can also exchange charge with the container, such that the net charge of the beads in the granular crystal is not zero. The overall net charge on the beads and container is not only determined by their relative position on the triboelectric series but also by the number of beads, their ratio, their geometry, and their kinetics, illustrating that the results can not be explained merely by the empirically established triboelectric series.^24^

Lately, Grzybowski’s group reported the formation of these granular crystals on supporting conductive substrates and showed that induced dipole and multipole interactions between the particles and the conductive substrate account for the stability of these granular structures.^23^ Interestingly, larger crystal structures were obtained when a second conductive substrate was placed on top of the setup without touching the beads, i.e., charge transfer with the top plate was excluded (cf. Figure 3b). This result implies that the electrostatic assembly of these granular crystals originating from the tribocharging of the system is not purely driven by the Coulomb interactions but also by the polar interactions *F*_pol_ ∝ *R*^3^ induced by the image charges located in the conductive substrates. Additionally, applying an electric field across the two conductive substrates induces dipoles on the particles which leads to openings in the crystal structures. However, these openings are reversible as soon as the electric field is switched off, implying that the assembled granular crystals can be manipulated in a controlled manner (cf. Figure 3c).^23^

The formation of granular crystals using electrostatic self-assembly is not restricted to spheres but could also be applied to obtain crystals of other millimeter-sized objects such as cubes (cf. Figure 3d) or rods by mechanical agitation.^22^

The aforementioned discussion on granular crystals may give the impression that agitation can easily be adapted to attain similar structures with micro- or nanoparticles. It is precisely the opposite, as significantly more energy should be applied to mobilize these particles with *Bo*_g_ > 1, particularly when agglomerated. The latter is supported by a study performed by Jimidar et al. in which peak accelerations of approximately 30*g* were required to mobilize the agglomerated monodisperse silica or polystyrene particles with diameters ranging between 3 and 10 μm on bare and fluorocarbon-coated silicon substrates.^12^ However, even at these accelerations, the strongly agglomerated hydrophilic silica particles would not fully mobilize compared to the weakly agglomerated hydrophobic polystyrene particles, as the cohesive interactions among the former are substantial compared to the polystyrene ones. On the other hand, the similarity between both types of particles is that they self-organize into monolayers on various substrates. The results are explained by accounting for adhesion and friction forces among the contacting bodies. Additionally, the study found compelling evidence using KPFM measurements that, due to the tribocharging mechanism, the adhesion between the microspheres and fluorocarbon-coated substrate was enhanced, promoting the formation of monolayers. The fluorocarbon-coated substrates locally gained electrical charge, while the polarity of only a few particles reversed after agitation (cf. Figure 3e), implying that charge exchange occurred between the microspheres and the substrate. In contrast to the ordered crystals obtained with millimeter-sized spheres,^22−24^ the monolayers comprising the microspheres were highly disordered, as confirmed by analyzing their morphology using the Voronoi approach. The different symmetry structures within the monolayers can possibly be explained by electrostatic repulsion between neighboring particles. Another reason may be that the particles’ kinetic energy was insufficient to overcome the adhesion forces, i.e., particles were stuck on the substrates, implying that it is plausible that higher frequencies and amplitudes were required to achieve ordered crystal structures.

Triboelectrification can also be exploited to direct the assembly of objects into nonclosely packed structures, as has been done elegantly by Wang et al.^25^ They obtained arbitrary, predesigned patterns of millimeter-sized nylon beads on a Teflon-coated, templated copper electrode (see Figure 3f). The nylon beads were precharged by shaking them in a Teflon container, while the Teflon coating was charged by rubbing it using aluminum foil. Consequently, the nylon beads were positively charged, while the Teflon coating was negatively charged, as predicted by the triboelectric series. However, positive charges were induced in the grounded copper electrode, screening the negative charge on some parts of the Teflon coating. After gently shaking the beads across the surface, the assembly strategy allows for trapping the positively charged beads solely on the remaining negatively charged regions on the Teflon coating, i.e., an electrostatic attraction emerged between the beads and the zones where the copper electrode was not present underneath the Teflon coating. Note that by tilting the system, excess millimeter-sized beads would just easily roll off the surface due to the gravity force. The applied assembly strategy here is reminiscent of the photocopying technique, which uses the electrostatic attraction of toner particles on a drum.

Next to agitation, manual rubbing is another type of mechanical-based techniques to separate agglomerates into single particles. A recent study applied this method to achieve assembled structures of agglomerated 10 μm silica particles on fluorocarbon-patterned substrates using a polydimethylsiloxane (PDMS) sheet.^19^ Note that, generally, surfaces are coated with fluorocarbon to eradicate capillary forces, decreasing the overall adhesion of particles. It was serendipitously observed that the hydrophilic silica microspheres adhered to the hydrophobic fluorocarbon patterns instead of the hydrophilic uncoated zones on the substrates. Despite the circular rubbing motion, the silica particles self-organized into nonclosely packed monolayer structures, matching the geometry of the fluorocarbon features on the substrates as shown in Figure 3g. By probing the fluorocarbon coating and particles using KPFM, it was concluded that they acquired opposite polarity, enhancing the adhesion of particles due to the electrostatic attraction.

In contrast to the study of Wang et al.,^25^ which leverages gravity to remove the excess particles by simply tilting the substrate, the excess microspheres in the above rubbing study could not be removed in this manner as the adhesion force between particles and uncoated parts of the substrates was considerably stronger than gravity. However, these excess particles could be removed from the uncoated parts by blowing pressurized nitrogen gas, while only a few assembled particles were removed from the fluorocarbon coating. These results show that due to the tribocharging mechanism, the induced electrostatic attraction significantly promotes the adhesion between the particles and the coating such that the particles can withstand the manual rubbing motion and the force exerted by the flow of nitrogen gas.

Compared to mechanical agitation, manual rubbing intrinsically allows for applying substantial shear forces to separate agglomerates into single particles and move them over larger areas. In addition, the assembled structures are attained within a minute (±15 rubbing strokes), whereas agitation takes at least 15 min to obtain large areas covered with monolayers. However, a significant drawback of the manual rubbing method is that the rubbing tool, e.g., the PDMS slab, also interacts with particles and substrate, corresponding to a three-material tribocharged system. Consequently, manual rubbing cannot investigate the pure interaction between the beads and the underlying substrate in contrast to mechanical agitation. In addition, the manual rubbing method is more susceptible to human imprecision and minute differences in applied force.

A key finding of the performed manual rubbing study is that the quality of the assembled monolayers was sensitive to air moisture and the supporting substrate material. The amount of water absorbed on surfaces changes with the relative humidity, possibly affecting the degree of tribocharging and concomitantly electrostatic attraction, as already reported by others.^3,6^ Another unexpected but significant finding was that the underlying substrate plays a crucial role in the interfacial charging behavior of the fluorocarbon coating with the particles. This remarkable discovery was first reported by Siek et al.^13^ Their study provides compelling evidence that the charge acquired on the polymer surface due to tribocharging is affected by the induced image charges in the supporting substrate, influencing the charge acquired. The thickness of the polymer surfaces varied up to 1 μm.

These examples collectively highlight the rich complexity of tribocharging emerging from mechanically induced friction between dissimilar materials. By perfectly balancing the electrostatic forces with other relevant surface interaction forces at that length scale, one can obtain a wide variety of electrostatically assembled patterns of dielectric objects. However, manipulating these interaction forces is much more challenging for microparticles than for larger beads as cohesion forces tend to dominate the interactions among fine powder particles or between those particles and substrates. Therefore, systematic studies on the dry electrostatic assembly of colloids are lacking, even though there is a myriad of colloidal particle types to be explored. For example, Locatelli et al. showed that a variety of particle arrangements could be achieved when tuning the interactions of heterogeneously charged (patchy) colloids on patterned substrates using Monte Carlo simulations.^28^

## 5 Granular and Similar Materials Charging

Up to now, we have elaborated on the tribocharging involving objects with different material compositions. However, particle tribocharging can not be addressed without shedding light on a highly counterintuitive problem: the tribocharging of identical materials. The latter is particularly the case in everyday granular charging events, e.g., sand storms, volcano explosions, and collisions of cosmic grains in early proplanetary formation.^29^ The understanding of granular charging is also relevant to engineering applications,^30^ such as fluidized beds, the nonuniform blending of pharmaceutical powders, and space explorations.

**Figure 4:**
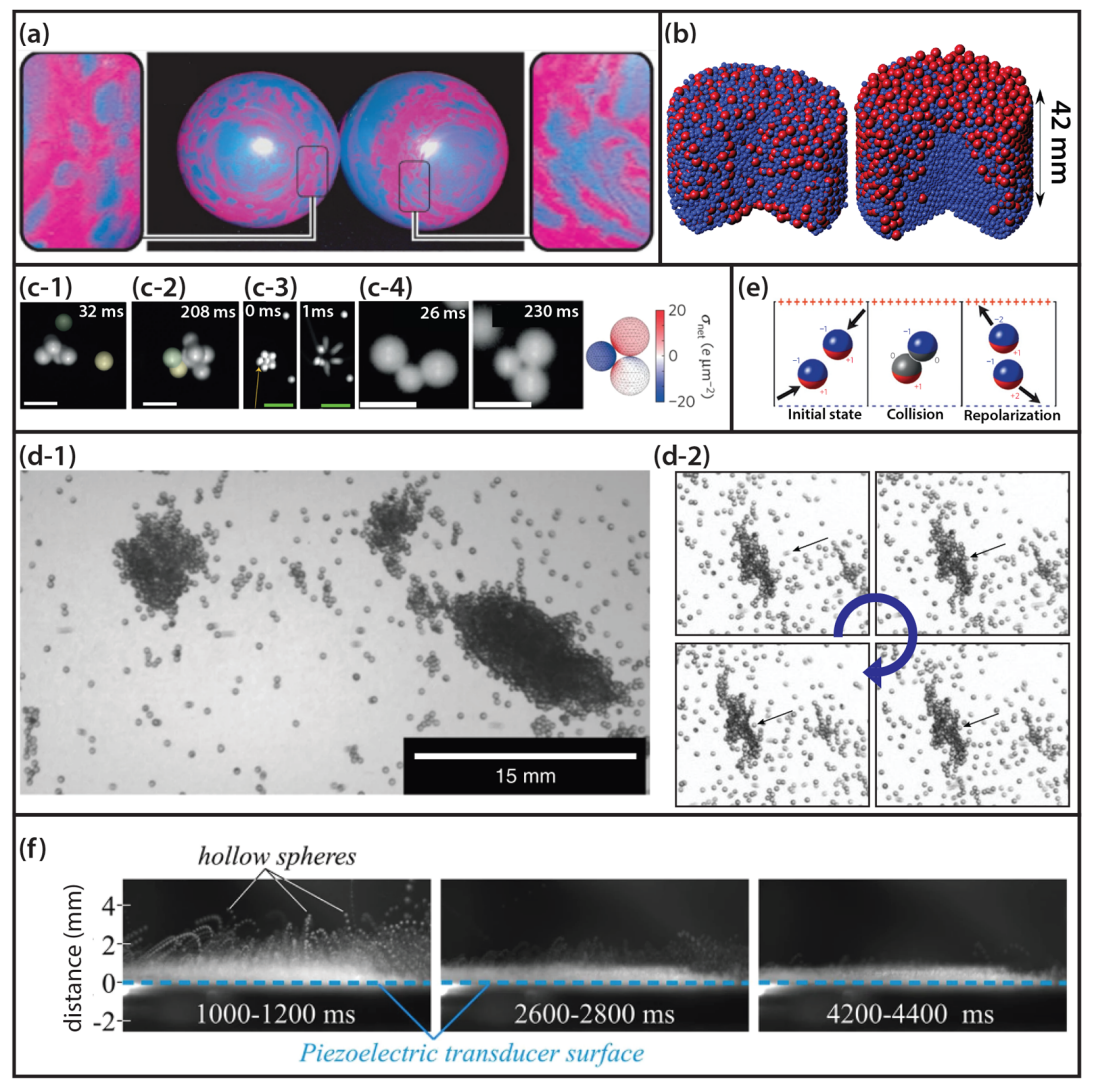
(a) Emerging charge patterns on two identical latex balloons after rubbing them against each other. (b) Segregation of an agitated bidisperse mixture (red = big, blue = small) of PTFE spheres at (left) a RH = 50%, and (right) a RH = 1000%. Time-lapse of (c-1, c-2) a growing, or (c-3) disruption of a cluster after microparticles collided against them. (c-4, left) Dynamic formation of a stable triangular structure comprising two large particles and one small particle. (c-4, right) Possible charge distribution producing these triangular structures. (c) Scale bar: 500 μm (white), and 1 mm (green). (d-1) Precharged agglomerates entering the Bremen tower. (d-2) Impact of a single granule (marked by the black arrow) against a large cluster. (e) Charging of uncharged identical particles in an external electrical field. (f) Time-lapse of vibrated hollow microspheres in the Bremen tower. (a) Reproduced with permission from ref (31). Copyright 2008 Europhysics Letters Association. (b) Reproduced with permission from ref (32). Copyright 2017 Royal Society of Chemistry. (c) Reproduced with permission from ref (33). Copyright 2015 Springer Nature. (d) Reproduced with permission from ref (34). Copyright 2019 Springer Nature. (e) Reproduced with permission from ref (35). Copyright 2010 Springer Nature. (f) Reproduced with permission from ref (36). Copyright 2017 Springer Nature.

The charging of identical materials after contact has been studied extensively but is certainly not limited to particles. It is reported that an asymmetry in contact geometry or size leads to tribocharging of identical surfaces. However, the same material tribocharging phenomenon starts to become more mysterious when completely identical materials with the same contact geometries break their surface charge symmetry as soon as they are released. After contact, a charge pattern is observed on the surface, e.g., on two symmetrically rubbed balloons (cf. Figure 4a), or PDMS sheets after conformal contact, implying that it is plausible that local surface heterogeneities or strain fluctuations contribute to charge transfer among the bodies.^31^ Surprisingly, charge transfer was not resisted by the Coulomb force as more charge transfer occurred in one direction when the number of repeated contacts was increased. This phenomenon has also been recently addressed by the Waitukaitis group by accounting for the spatial correlations in donor/acceptor properties in their model.^37^

The inevitable multiple collisions among similar beads add to the complexity of tribocharging in a granular system. Consequently, for bidisperse or polydisperse systems, large particles tend to charge positively, while small particles acquire a negative polarity. For example, a study performed by Schella et al.^32^ using a bidisperse system of millimeter-sized polymer beads confined in a container showed that two distinct millimeter-sized PTFE beads gained opposite polarity after shaking this bidisperse system vertically, segregating the bed of particles in the container. Note that there is a chance, albeit minimal, that the particles can collide with the container walls, but the charge on the particles is affected mainly by the particle–particle collisions. A key finding of the study was that the particles’ charge was lower when the humidity levels increased, while the segregation was suppressed at low humidity levels as shown in Figure 4b. In the latter case, the particles acquired more charge, such that the dominant electrostatic attraction among the distinct beads renders a mixed granular system. Thus, it is inferred from this study that various packing structures can be contrived using a mixture of granules by controlling the humidity and concomitantly the charge on the beads.

As mentioned, many multiple head-on collision events occur among identical grains in a granular system. At the moment of the collisions, the granules experience a repulsive collision force *F*_col_ ∝ *R*^3^. Note that the attractive gravitation force between two identical particles is *F*_grav_ ∝ *R*^4^, while the attractive cohesion forces are *F*_coh_ ∝ *R* (cf. section 2). The interplay between these short-range and repulsive forces can cause two particles to stick and form dimers that act as seeds in subsequent collisions to form larger agglomerates. Basically, if the attractive forces defy the collision force, particles will stick. Considering the dependency of the forces on the particles’ size, it is inferred that gravity becomes the dominant force for large body stickiness. In contrast, in the case of microparticles or colloids, the cohesive forces tend to become more significant than the collision force, promoting the formation of agglomerates.

Of course, the particle size does not paint the whole picture, as the collision force also pertains to the impacting bodies’ momentum and the coefficient of restitution (COR = ratio of relative velocity magnitude before and after the impact). The latter depends on the bodies’ mechanical properties, e.g., Young’s modulus, and yield stress.^16^ Despite the tangled interplay of several factors, the particles’ kinetic energy should surpass the cohesive energy resulting from the short-range forces for them to bounce.

However, a study executed in a tower with a dilute stream of monodisperse zirconium dioxidesilicate grains with a diameter of 274 μm showed that the long-range Coulomb and short-range induced polarization forces continuously attract particles between repeated collisions (Figure 4c-1).^33^ Consequently, the formation of clusters was enhanced (Figure 4c-2), but particles with sufficient kinetic energy could also disrupt already existing aggregates (Figure 4b-3). The particles with just a minimal size dispersion obtained a net charge distribution centered around zero, with some particles remarkably acquiring several millions of elementary charges *e* (1.6 × 10^–19^ C). In turn, these highly charged particles served as nucleation seeds by attracting lesser charged particles to form densely packed arrangements with multiple particles in contact (cf. Figure 4c-2). The stability of these extraordinary arrangements can be ascribed to the induced polarization forces. These forces are always significantly attractive for particles with a high dielectric constant, even when they carry a charge of the same polarity but with a prominent contrast in magnitude. Therefore, the induced polarization force is essential, as it enhances the likelihood of lattice structure formation at a close approach. The latter is particularly elucidated when experiments were performed with a bidisperse mixture of larges beads (326 μm) and smaller beads (251 μm) of the same material. As expected, the large particles were positively charged, and the smaller beads gained a negatively charged surface. Various stable electrostatically formed structures comprising large and small beads were formed, one of which is depicted in Figure 4c-4. It is observed that the two positively charged large spheres are attracted to each other to form a stable triangular structure with the small particle, which can be attributed to the induced polarization forces. Thus, colliding grains can turn into stable structures, so-called “granular molecules”, by attractive electrostatic interactions provided that their kinetic energy is minimized.

Similarly, the Wurm group observed the spectacular electrostatic driven formation of stable centimeter-sized agglomerates consisting of single monodisperse glass particles (434 μm) in the Bremen tower under microgravity conditions.^34^ Prior to releasing the particles in the tower, where an electric field is applied under microgravity conditions, the grains were precharged by shaking them in a container. Consequently, the grains were tribocharged and formed millimeter-sized agglomerates before entering the tower (cf. Figure 4d-1). Strikingly, these agglomerates remained intact even after colliding at speeds of a few decimeters per second. However, due to electrostatic interactions, the agglomerates could deform (cf. Figure 4d-2) or even grow to larger structures.

From a fundamental perspective, this result is highly significant as it demonstrates that millimeter-sized colliding entities can overcome the so-called bouncing barrier relevant to planetesimal formation and growth. Earlier studies reported that uncharged aggregates larger than a few millimeters beyond the bouncing barrier would collide but not stick and may even be fragmented into smaller agglomerates.

Another concept proposed for identical particle charging in particle clouds is the collision of dielectric spheres in the presence of an electric field without accounting for any conductive mechanisms on the particles or the environment. In this model, depicted in Figure 4e, two neutral dielectric spheres are polarized by an external electric field, and their charge gets neutralized at their point of contact. Subsequently, the particles are repolarized after the rebound, such that the particles acquire an opposite polarity.^35^ A major concern of this model, as truthfully acknowledged by the authors, is that these external electric fields are nonexisting in natural events.

On the other hand, Yoshimatsu et al. reported that external electric fields are not needed to polarize dielectric spheres.^36^ It is shown that the electric field generated by an infinitesimal charged bead is sufficient to polarize neighboring particles, charging them according to the same concept shown in Figure 4f. Surprisingly, the charge on agitated identical particles is amplified and increases exponentially. The latter is corroborated by agitating hollow microspheres with a diameter of ±140 μm also in the microgravity environment of the Bremen tower. From the results shown in Figure 4f, it can be inferred that the trajectory of the spheres leaving the particle bed was parabolic, implying that they only experienced an electrostatic force as gravity effects are nullified in the Bremen tower. In addition, as the acquired charge on the particles increased exponentially, and concomitantly the electrostatic attraction among particles, the levitation of particles from the agitation bed was inhibited.

To sum up, identical materials get tribocharged if an asymmetry exists between the bodies, which can be caused by substantial differences in size, geometry, small fluctuations in strain, surface charge densities, or other surface heterogeneities. These may arise from ambient conditions, such as humidity and environmental ionic species. It is also reported that even on hydrophilic materials water islands are present instead of a continuous layer water,^38^ which also may effect the local charging of identical materials. Additionally, several studies highlighted the significance of polarization forces c.q. induced dipoles, even between like-charged particles, making them indispensable when it concerns the charging of similar materials.^27^ Consequently, stable granular molecules or large aggregates can be formed, offering insight into the self-assembly of colloidal particles or the formation of planetoids.

## 6 Summary and Future Outlook

Spanning the last centuries, a plethora of scientists embarked on a journey to comprehend the omnipresent yet so mysterious, tribocharging phenomenon in which charge is exchanged at the interface between two bodies momentarily in contact. Rubbing balloons or grains using a PDMS sheet may be science’s most straightforward experiment to perform or demonstrate without using expensive equipment. However, as we have highlighted, tribocharging can already be affected by the slightest surface inhomogeneity, which may be considered the Achilles’ heel to arriving at a unifying mechanism explaining this manifestation of charge transfer present since the old days. The different charging mechanisms proposed to date elucidate that we have only scratched the surface of understanding electrostatic charging.

In the present contribution, we have aimed to marry the solid conceptual ideas from chemists, materials scientists and physicists, particularly those working on granular matter, on the tribocharging of different objects at various scales. Considering Richard Feynman’s quote: “there is plenty of room at the bottom”, we envision that these concepts could also be adopted for the dry assembly of crystals comprising microspheres or colloids. Plausibly, the latter can be realized by tuning the surface chemistry of the particles to reduce their tendency to stick on surfaces. Next, advances in contemporary polymer and colloidal chemistry allow synthesizing particles with shape anisotropy, e.g., rods, cubes, ellipsoids, and bespoke chemical functionality such as Janus particles or patchy colloids.^39,40^ In addition, these aspherical particles could also be utilized in granular matter studies as, in practice, particles may not be completely spherical. It is expected that the shape anisotropy will affect the surface charge distribution and, consequently, the induced dipoles and assembled structures. Since anisotropic particles are not readily available, systematic studies investigating shape effects on tribocharging at the micro- and nanoscales are lacking. Thus, large gains lie within grasp concerning our understanding of the shape anisotropy of particles or substrates on assembled structures.

Next, as inferred from the rubbing study performed using two identical balloons, a charge pattern existed on their contact area, implying that this is potentially also the case for colliding particles. Thus, instead of only having a net charge *q*, particles may carry charge patches, as well as water patches, on their surface. Consequently, these patches affect the attractive polarization between neighboring particles, which significantly contributes to the formation of stable electrostatically assembled structures. To gain more insight into the charge distribution on the colliding bead, one can perform, for example, KPFM measurements. However, quantitative data on the charge distribution will remain missing. Therefore, a clear impetus remains to develop methods to produce quantitative data with a nanoscale resolution to take a quantum leap in our understanding of charge transfer among identical materials.

The existing colloidal probe technique has rarely been employed to probe the electrostatic interaction between particles and substrates in the air. Although the acquired polarity of the surfaces can not be retrieved, we believe this technique can be leveraged more frequently to study the electrostatic interaction of particles on surfaces in air. This technique allows the study of tribocharging effects in a controlled manner by varying parameters, e.g., humidity, surface chemistry, load, and concomitantly contact area. These measurements may be relevant to the adhesion of dust particles on solar panels.

Finally, tribocharging encompasses such a rich complexity that unravelling it will be advantageous to the quest for manufacturing novel responsive materials, energy harvesters, wearable materials, more efficient solar panels, as well as our understanding of planet formation. We want to warn those entering the tribocharging field that the triboelectric series is not the holy grail but should be merely treated as a guideline in which direction charge transfer will occur. Moving forward, scientists across all disciplines must contribute to overcoming challenges in understanding the tribocharging of beads, such as synthesizing perfectly identical particles and designing novel experimental techniques that can generate quantitative data under highly controlled and reproducible conditions. These advancements should prevent any asymmetry when studying the charging of completely identical materials. Thus, exciting opportunities lie ahead regarding the dry assembly of structures comprising micro- and nanoparticles.
